# Factors Important in the Use of Fluorescent or Luminescent Probes and Other Chemical Reagents to Measure Oxidative and Radical Stress

**DOI:** 10.3390/biom13071041

**Published:** 2023-06-26

**Authors:** Peter Wardman

**Affiliations:** Formerly of the Gray Cancer Institute, Mount Vernon Hospital/University of Oxford, UK; peterwardman@btinternet.com

**Keywords:** free radicals, oxidative stress, reactive oxygen species, ROS, superoxide radicals, nitrosative stress, fluorescent probes, luminescent probes, rate constants, pulse radiolysis

## Abstract

Numerous chemical probes have been used to measure or image oxidative, nitrosative and related stress induced by free radicals in biology and biochemistry. In many instances, the chemical pathways involved are reasonably well understood. However, the rate constants for key reactions involved are often not yet characterized, and thus it is difficult to ensure the measurements reflect the flux of oxidant/radical species and are not influenced by competing factors. Key questions frequently unanswered are whether the reagents are used under ‘saturating’ conditions, how specific probes are for particular radicals or oxidants and the extent of the involvement of competing reactions (e.g., with thiols, ascorbate and other antioxidants). The commonest-used probe for ‘reactive oxygen species’ in biology actually generates superoxide radicals in producing the measured product in aerobic systems. This review emphasizes the need to understand reaction pathways and in particular to quantify the kinetic parameters of key reactions, as well as measure the intracellular levels and localization of probes, if such reagents are to be used with confidence.

## 1. Introduction

While most papers in this Special Issue focus on modified endogenous biological molecules as markers of oxidative, nitrosative or other changes initiated by free radicals, such damage has also been routinely assessed using exogenous, xenobiotic molecular probes. Thus, as described below, a reduced fluorescein dye has been widely used to detect the production of (usually unspecified) ‘reactive oxygen species’ in biological material or biomolecular models—astonishingly, in over 6000 studies to date—despite the obvious shortcomings discussed below. Hence, it is important not to take a too narrow or literal viewpoint in considering ‘biomarkers’ of oxidative stress and radical damage.

There will often be competing pathways in the production of endogenous biomarkers of oxidative or other radical damage, with the yield of biomarker reflecting not only the production of radical reactants but also the concentrations and availability of potentially protective substances such as thiols, ascorbate or phenolic antioxidants (for example). Similarly, the ‘signal’ reflecting chemical change in exogenous markers or probes, whether measured concentrations or image intensity (e.g., in fluorescence readers or microscopy), may reflect not only the extent of the production of radicals or oxidants but also the involvement of competing reactions. The outcome of such competing processes will be defined by the Law of Mass Action, and so the important characteristics will be the product of concentration *and rate constant* for all the competing reactions. Hence, whether damage is assessed by endogenous biomarkers or by added chemical probes, chemical kinetics is central to an informed assessment of the damage pathways, and the availability of rate constants for specific reactions involving free radicals (in particular) and potential biomolecular targets (or reasonable models for them) is central to an informed discussion about competing pathways in free-radical biology. Of course, the intracellular concentrations of reactants *or* probes are equally important, and cellular heterogeneity is a daunting challenge to the accurate chemical modelling of biological pathways, as discussed elsewhere in the context of radiation damage [1]. As an example, ‘molecular crowding’ can modulate reaction rates (or effective rate constants), as illustrated recently [2]. As a first step, though, analyzing potential competing reactions by simple homogenous competition kinetics is far better than nothing, even if, ultimately, more sophisticated approaches are desirable, such as applying Monte Carlo numerical simulation methods to a heterogenous model of cellular compartments.

This review aims to illustrate the importance of kinetic factors in using chemical probes for radical-initiated damage, although the concepts apply equally to discussing the production of endogenous biomarkers. It will also help readers to access sources of pertinent kinetic information in both contexts. Kinetic information (rate constants) can be estimated by measuring the final product yields analyzed by competition kinetics in model systems where a reference reaction has been characterized separately, but the direct observation of reactive intermediates—radical or product—is usually the most reliable. This involves the production of sufficient radicals in a time significantly shorter than the reaction timescale to follow the reactions and enable detection, e.g., by kinetic spectrophotometry. The technique of flash photolysis, introduced around 1950, made this possible for photochemically initiated reactions. The analogous technique of pulse radiolysis, introduced a decade later, is much more relevant for free-radical reactions, since ionizing radiation generates free radical pathways which, in the case of water as a solvent, can be easily manipulated to monitor reactions of specific radicals of biological interest. Hence, radiation chemistry has provided, as a ‘spinoff’, much kinetic information concerning reactions involving free radicals and biomolecules. It is not necessary to understand the complexity of radiation damage to appreciate the application of the specialized techniques to general redox chemistry [3].

It is not intended here to give an extensive overview of the chemistry and/or methodology of markers and/or probes for ‘reactive oxygen species’ or oxidative damage, which has recently been reviewed with specific recommendations [4]. The present author reviewed the chemistry of fluorescent and luminescent probes for oxidative and nitrosative stress in some detail [5], and numerous more recent reviews in this area updated and expanded the earlier survey [6,7,8,9,10,11,12,13,14,15,16,17,18,19,20,21,22,23,24,25,26,27,28,29,30,31,32,33,34,35,36,37,38,39,40,41,42,43,44,45,46,47,48,49]. Rather, the intention here is to illustrate the importance of understanding the reaction pathways involved in the use of these probes and, especially, the importance of kinetics in controlling these pathways.

We first have to consider the terms used in labelling these probes; following this, a discussion of the chemistry of some of the most commonly used probes serves as a ‘template’ or ‘worked example’ against which other probes can be assessed. Finally, the questions which all users of chemical probes should ask themselves before commencing on a study are briefly summarized.

## 2. ‘Oxidative Stress’ and ‘Reactive Oxygen Species’ (‘ROS’): Their Definitions (or Lack of)

If we are to discuss sensibly probes for chemical species, then we need to be clear about the molecules involved. While the term ‘oxidative stress’ is reasonably well understood and quite well defined [50,51,52,53], the related but not synonymous term ‘reactive oxygen species’ and its acronym ‘ROS’ are mere ‘catch-all’ phrases lacking usefulness because of their vague, all-encompassing nature. Indeed, experts in free-radical biology are increasingly warning about the indiscriminate use of ‘ROS’. Thus, it was recommended: ‘The use of ROS or RNS should be … only when it is clearly stated that the species is unknown or one of several implicated molecules without certainty’ [54]; it was commented: ‘The ubiquitous use of these terms seems to provide a screen to hide the detailed chemistry of these species’ [55]. Another group of experts noted: ‘Reactive oxygen species’ (ROS) is a generic term that defines a wide variety of oxidant molecules with vastly different properties and biological functions … The generic term ROS should not be used to describe specific molecular agents’ [43]. The first recommendation of an expert review group [4] was ‘… wherever possible, the actual chemical species involved in a biological process should be stated, and consideration given to whether the observed effect is compatible with its reactivity, lifespan, products generated and fate in vivo’.

The difficulty in beginning to assess the reactions of vague ‘ROS’ in kinetic terms is apparent if we compare the rate constants for reactions of the common biological antioxidant, the thiol, glutathione (GSH), with specific radical or molecular oxidants, all containing oxygen, at physiological pH. Thus, the rate constants (units of M^−1^ s^−1^) for reactions of GSH with different ‘ROS’ span over *ten orders of magnitude*, with the rate constants for ^•^OH (~1 × 10^10^ [56]), HOCl (~1 × 10^8^ [57]), NO_2_^•^ (~2 × 10^7^ [58]) and CO_3_^•−^ (~5 × 10^6^ [59]) being several orders of magnitude higher than those for reactions of O_2_^•−^ (~2 × 10^2^ [60]) and H_2_O_2_ (~9 × 10^−1^ [61]).

In the context of chemical probes for oxidizing radicals derived from biomolecules, it should also be noted that radicals centered on *sulfur* (thiyl radicals, RS^•^, and not normally viewed as ‘reactive oxygen species’) can oxidize targets directly: the mid-point reduction potential of the couple GS^•^,H^+^/GSH is ~0.90 V at pH ~7 [62], only ~0.04 V lower than that of the tyrosine phenoxyl radical, TyrO^•^,H^+^/TyrOH [63]. Thiols are often viewed as protective antioxidants, but they do have the *potential* to *elevate* ‘ROS’/oxidative stress following the chemical ‘repair’ of diverse radical sites via the sequence of reactions [64,65]:

radical damage + RSH → ‘repaired’ (or modified) damage + RS^•^

RS^•^ + RS^−^ ⇌ (RSSR)^•−^

(RSSR)^•−^ + O_2_ → RSSR + O_2_^•−^.

However, ascorbate (AscH^−^) can disrupt this pathway by intercepting thiyl radicals [66]:

RS^•^ + AscH^−^ → RSH + Asc^•−^,

but it is important to note that many in vitro cell culture models lack ascorbate and so are poor models for tissues. The potential reaction pathways of thiyl radicals in cells are multiple and complex [67,68,69] and include the catalysis of the *cis*/*trans* isomerization of unsaturated lipids [70,71,72,73]. Since thiyl radicals can form reactive thiylperoxyl (RSOO^•^) and sulphonyl radicals in the presence of oxygen [74,75], thiol radical chemistry should be included in any discussion of ‘ROS’.

It has also long been recognized that ‘ROS’ should not be viewed in isolation, in particular, reactive nitrogen-based oxidants include peroxynitrous acid/peroxynitrite (ONOOH/ONOO^−^, from the reaction between ^•^NO and O_2_^•−^), which in turn can source ^•^OH and ^•^NO_2_ as well as carbonate radicals (CO_3_^•−^) [76,77]. CO_3_^•−^ and NO_2_^•^ are fairly powerful oxidants: the reduction potentials of the couples CO_3_^•−^/CO_3_^2−^ and ^•^NO_2_/NO_2_^−^ are ~1.57 and 1.04 V, respectively [78], higher than the midpoint potentials at pH 7 of the radical/reductant couples of tyrosine, glutathione or ascorbate [3]. Hence, the spectrum of radical oxidants potentially reactive towards chemical probes is quite wide—and of course the non-radical oxidants H_2_O_2_ and HOCl cannot be ignored, particularly since H_2_O_2_ can form oxidizing intermediates in peroxidase chemistry or upon reaction with cytochrome *c*, as noted below. Overall, then, a whole battery of oxidants are *potentially* reactive towards *some* chemical probes for ‘ROS’ or oxidative stress, presenting a major challenge to the informed use of probes.

## 3. Dichlorodihydrofluorescein (DCFH_2_): By Far, the Most Widely Used—And Certainly the Most Abused—Probe for ‘ROS’ or Cellular Oxidative Stress

It is sensible—and instructive—to illustrate the importance of mapping reaction pathways of molecular probes and to characterize them kinetically by discussing the chemistry of the most widely used probe for oxidative events associated with free radicals in biology, not least because the reactivity of this probe has been kinetically characterized quite well. However, as discussed below, there remain many gaps in our knowledge, and the results of any study using this or similar probes must continue to be viewed with skepticism.

**Figure 1:**
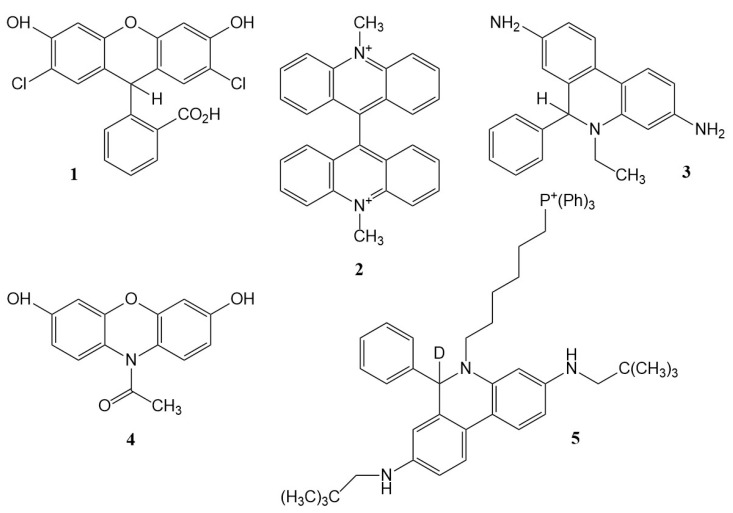
Structures of the probes discussed: **1**, dichlorodihydrofluorescein (DCFH_2_); **2**, lucigenin; **3**, hydroethidine (HE); **4**, Amplex Red; **5**, ‘MitoNeoD’.

Assays based on the fluorescence of a probe or its product are both sensitive and facilitate imaging. Fluorescein is perhaps the archetypical fluorophore, and its reduced form (dihydrofluorescein) is a non-fluorescent or ‘leuco’ dye. The variant 2′,7′-dichlorodihydrofluorescein (DCFH_2_, Figure 1, **1**) is the most commonly used probe for vague ‘ROS’ or general oxidative damage in biology associated with free radicals, with the fluorescent product dichlorofluorescein (DCF) measured; an appropriate PubMed search revealed almost 7000 ‘hits’ in May 2023 for papers referencing these dyes. Even accounting for the papers which simply mention or review the use of DCFH_2_ or related probes, it seems likely that well over 6000 studies have utilized such probes. Despite well-known shortcomings, which have been repeatedly stressed by numerous authors, e.g., [5,6,8,9,13,19,23,24,27,30,54,55,79,80,81,82,83,84,85,86,87,88,89], these probes continue to be widely recommended and used in many investigations, e.g., [90,91,92,93,94,95], including a recent study that attracted quite widespread press coverage [96].

There are three basic problems with this and similar ‘leuco’ dyes such as the chloromethyl analogue and other variants promoted by manufacturers; while DCFH_2_ is the best characterized, it seems likely that all exhibit many of the problems discussed below. First, oxidation may reflect the level of the catalyst rather than that of the oxidant; second, the rate of oxidation by radicals varies widely, and radicals other than ‘ROS’ react rapidly with the probe; and third, the radical intermediate produced during oxidation—either directly by radicals or by a catalyst activated by H_2_O_2_—reacts rapidly with oxygen *to generate superoxide radicals*.

Consider first oxidation by non-radical species, of which H_2_O_2_ is the most studied. For a start, the direct reaction between H_2_O_2_ and DCFH_2_ is very slow, and the reaction is most unlikely to compete with the destruction of H_2_O_2_ by cellular peroxidases, peroxiredoxins, etc.: oxidation by H_2_O_2_ requires a *catalyst*, as shown in early studies with DCFH_2_ or analogues where horseradish peroxidase (HRP) or hematin was used [97], and the signal measured, therefore, may reflect the level of catalyst rather than (or as well as) the level of H_2_O_2_ generation. Fluorescent probes for catalytic iron(II) were developed [34]. Another potent catalyst is cytochrome *c* [98], very important because it is released from the mitochondria during apoptosis. Further, diverse hydroperoxides can substitute for H_2_O_2_ in the catalyzed oxidation of DCFH_2_ [79]. The peroxidase- or cytochrome *c*-catalyzed oxidation is also subject to interference by antioxidants, including uric acid, ascorbate and α-tocopherol [79], as well as NADH and thiols [99]. Overall, then, associating fluorescence with changes in H_2_O_2_ production rather than with the levels of catalyst or antioxidants is difficult, and confusion with both apoptotic pathways and hydroperoxide formation rather than H_2_O_2_ is quite possible.

Other non-radical oxidants have been investigated in this context: hypochlorite reacts inefficiently with DCFH_2_ [100]; peroxynitrite (ONOO^−^) is much more efficient, but kinetic studies [101] showed this probably reflects radical oxidation involving NO_2_^•^, ^•^OH and/or CO_3_^•−^ radicals, decomposition products of peroxynitrite under physiological conditions [76,77].

The rates of oxidation of DCFH_2_ by radical oxidants vary widely: superoxide has very low reactivity, while ^•^OH radicals react at the diffusion-controlled limit (rate constant *k* ~1.3 × 10^10^ M^−1^ s^−1^; intermediate reactivity is seen with NO_2_^•^ (*k ~*1.3 × 10^7^ M^−1^ s^−1^) and CO_3_^•−^ (*k* ~2.6 × 10^8^ M^−1^ s^−1^, pH 8.2)) [102]. (Unless otherwise indicated, the rate constants here refer to physiological pH; the pH value can be important, since DCFH_2_ dissociates with p*K*_a_ values estimated as ~7.9 and 9.2 [102], and oxidants may show pH-dependent reactivity. NO_2_^•^, for example, may oxidize a phenolate species considerably faster than the corresponding phenol [103,104].) Sulfur-centered radicals (RS^•^, thiyl) oxidize DCFH_2_ with rate constants of ~3.7 × 10^7^ (GSH) or 1.7 × 10^7^ (cysteine) M^−1^ s^−1^ [85]; since such radicals are commonly produced in diverse radical ‘repair’ reactions (e.g., the donation of H to carbon-centered radicals, as noted above), the lack of specificity of DCFH_2_ towards oxygen-based radicals is obvious. Note, however, that the involvement of thiols in probe chemistry is more complex than the direct oxidation of DCFH_2_ by thiyl radicals: the oxidized probe DCF has a phenolic moiety and is a substrate for peroxidases, the resulting phenoxyl radical DCF(–O^•^) oxidizing GSH (as well as NADH and ascorbate), generating superoxide radicals via further reactions [81]:

DCF + oxidant → DCF(–O^•^)

DCF(–O^•^) + GSH (or NADH) → DCF + GS^•^ (or NAD^•^)

GS^•^ + GSH ⇌ (GSSG)^•−^

(GSSG)^•−^ + O_2_ → GSSG + O_2_^•−^

NAD^•^ + O_2_ → NAD^+^ + O_2_^•−^.

The oxidized probe also reacts rapidly with some radicals, e.g., the rate constant for the reaction of CO_3_^•−^ radicals with DCF is almost identical to that for the reaction of DCFH_2_; however, DCF is much less reactive towards NO_2_^•^ than is DCFH_2_ [105].

By the rule of spin conservation, the oxidation of DCFH_2_ to the fluorescent product DCF by radicals *must* produce a radical intermediate, DCFH^•^. Further, the catalyzed oxidation of DCFH_2_ also proceeds via this radical intermediate. In landmark studies [80,81,83], it was shown that this radical also reacts with oxygen to produce superoxide:

DCFH^•^ + O_2_ → DCF + O_2_^•−^ (+ H^+^)

leading the authors to describe such probes for oxidative stress as a ‘self-fulfilling prophesy’ [83]. A direct observation of the reaction of DCFH^•^ with O_2_ showed *k* ~5.3 × 10^8^ M^−1^ s^−1^ [102]. The rate of the possible competing reaction:

2 DCFH^•^ → DCFH_2_ + DCF

is pH-dependent around physiological pH because of prototropic equilibria, but the effective rate constant is 2*k* ~2.8 × 10^8^ M^−1^ s^−1^ at pH 7.4. Since the two rate constants are of the same order, and it is easily calculated that in most biological systems, the concentration of O_2_ far exceeds that of DCFH^•^ at a steady state, superoxide formation seems an inevitable consequence of DCFH_2_ oxidation, whether by (catalyzed) peroxides or directly by radicals [102].

The extent of DCFH_2_ oxidation by both H_2_O_2_ (or hydroperoxides) and radicals will reflect competition kinetics (the Law of Mass Action), since there are obviously competing pathways for the species to react, but this presents an immediate difficulty: the reaction pathways reflect the products of rate constant and concentration, but hardly any users of these probes have measured the intracellular concentration of DCFH_2_. Usually, the diacetate derivative is used, relying on cellular esterases to cleave the diacetate and relying on the resulting negative charge on the dissociated carboxylic acid to trap the probe intracellularly. In the author’s Laboratory, L K Folkes measured ~300 µM DCFH_2_ as the *average* intracellular concentration after incubating hamster fibroblasts in cell suspension (5 × 10^6^/mL) at pH 7.4 with DCFH_2_ diacetate (10 µM) for 15 min at 37 °C [102].

There will be intracellular concentration gradients typical of a weak acid, as well as lipid/water partitioning of both probe and product [106]; this study reported DCFH_2_ was ‘totally partitioned into the octanol’ using octanol/water 1:1 at pH 6, while DCF had a partition coefficient of 2:1 (the octanol:/water partition coefficient of DCFH_2_ at an unspecified pH was quoted elsewhere as 2.62 [107], but it will be pH-dependent because of prototropic equilibria [102]). There may also be leakage of product into the extracellular medium [108]. Further, the nature of the culture medium must be considered, including the presence of catalytic metals, antioxidants, pH indicators, etc. [109,110].

Considering, as an example, competing reactions between CO_3_^•−^ and either DCFH_2_ or GSH present together, the rate constant for the reaction of CO_3_^•−^ and GSH is ~5.3 × 10^6^ M^−1^ s^−1^ at pH ~7 [59], i.e., ~50–fold lower than that for the reaction between CO_3_^•−^ and DCFH_2_, but the intracellular concentration of GSH is typically around 10-fold higher than that of DCFH_2_ *if* the concentration of the latter is similar to that in the measurements with hamster cells described above. (Ascorbate reacts with CO_3_^•−^ ~260 × faster than does GSH [111].) Nominally, just considering the one competing reaction with GSH, the probe will ‘capture’ a good fraction—but not all—CO_3_^•−^ radicals, but since other free thiols are present along with other targets for CO_3_^•−^ radicals (protein thiols, tyrosine residues, etc., and ascorbate in tissues), it seems likely that the probe is often not being used in ‘saturating’ conditions, making quantitation difficult. This is more clear-cut if we consider competing reactions of NO_2_^•^ radicals. The rate constants for the oxidation of GSH or cysteine at pH 7.4 by NO_2_^•^ are ~2 or 5 × 10^7^ M^−1^ s^−1^, respectively [58], i.e., both higher than that for the reaction of NO_2_^•^ with DCFH_2_ (~1.3 × 10^7^ M^−1^ s^−1^). Hence, ~0.3 mM DCFH_2_ will ‘pick up’ only a very small fraction of any NO_2_^•^ generated. The total ‘scavenging capacity (Σ*k*[scavenger]) for ^•^OH radicals in mammalian cells has been estimated as ~8 × 10^8^ s^−1^, compared with the corresponding value for ^•^OH reacting with 0.3 mM DCFH_2_, i.e., ~4 × 10^6^ s^−1^; so, clearly it is most unlikely that DCFH_2_ (and indeed most probes at realistic intracellular concentrations) can intercept a significant fraction of ^•^OH radicals.

Kinetic competition may also be exhibited between the activated peroxidase intermediate (such as HRP Compound I) reacting with either the probe or endogenous antioxidants; so, for a full understanding of probe chemistry, quite extensive studies must be undertaken. These are likely to involve several sophisticated techniques; in this case, electron paramagnetic resonance spectroscopy to identify reaction intermediates, stopped-flow rapid mixing to measure the rates of non-radical reactions, flash photolysis to monitor reactions of excited states (see below), high-performance liquid chromatography to measure intracellular uptake, preferably in specific organelles, and (especially) pulse radiolysis to generate specific ‘ROS’ in known amounts and monitor their reactions in real time.

Even leaving aside the obvious contraindication of the generation of one specific ‘ROS’ (superoxide radical) via the obligate involvement of an oxygen-reactive radical in the formation of the fluorescent product, without measurements of *both* the rate constants of the reactions of putative reactants with a probe *and* the estimates of the intracellular (or better, intra-organelle or localized) concentrations of the probe, it is obvious that users are working in the dark in seeking to quantify reactive oxidants using such probes. Actually, users *had* best work in the dark, because a further complication is that visible light can generate an excited state of DCF, quenched by cellular reductants such as GSH or NADH (and doubtless other reactants such as ascorbate) to generate DCFH^•^ and hence O_2_^•−^ [112]. Photooxidation of Amplex^®^ Red has also been reported, once again involving the *production* of superoxide radicals [113].

## 4. Some Kinetic Studies of Other Chemical Probes for Oxidants and Biological Radicals

DCFH_2_ is not the only ‘ROS’ probe to be a ‘self-fulfilling prophesy’. As discussed in more detail previously [5], the bis-*N*-methyl acridinium salt, lucigenin (LC^2+^, Figure 1, **2**), is reduced to a radical LC^•+^, probably by flavoprotein reductases, and exhibits chemiluminescence on reaction of this radical intermediate with superoxide radicals via an unstable intermediate producing an excited state of *N*-methylacridone [114]. However, the obligate radical intermediate LC^•+^ reacts with oxygen *to generate superoxide* [115,116] with a rate constant of ~3 × 10^6^ M^−1^ s^−1^:

LC^•+^ + O_2_ ⇌ LC^2+^ + O_2_^•−^

and since the reduction potential of the LC^2+^/LC^•+^ couple is –0.28 V vs. NHE, the equilibrium constant of the reaction is ~50; the reverse reaction producing LC^•+^ from superoxide must have a rate constant of ~6 × 10^4^ M^−1^ s^−1^ and is unlikely to compete with the reaction of O_2_^•−^ with superoxide dismutase in experiments involving loading LC^2+^ into mammalian cells [117]. Again, despite such an obvious contraindication, the probe is still being used: thus, in a recent study ‘… superoxide production in the brain, heart tissue, and aorta was measured using lucigenin-enhanced chemiluminescence’ [118].

Even if repeated warnings about the shortcomings of commonly used probes continue to be ignored, some groups *have* exhibited a clear understanding of the problems; the collaboration between the Institute of Applied Radiation Chemistry at Łodz University of Technology and the Medical College of Wisconsin has been particularly fruitful (e.g., [9,27,30,39]), and considerable progress is being made in the kinetic characterization of some probes. Some examples are outlined very briefly below. Nonetheless, it is not at all encouraging that, while a widely consulted *Handbook of Fluorescent Probes* (see: https://www.thermofisher.com/uk/en/home/global/forms/mp-handbook-download-request-form-2014.html, accessed on 27 May 2023) has a 27-page chapter devoted to ‘Probes for Reactive Oxygen Species, Including Nitric Oxide’, the terms ‘rate’ or ‘rate constant’ are both conspicuously absent.

Progress is also illustrated by a discussion of the extent of intercepting oxidants using the more recently introduced boronate probes for nucleophilic oxidants, which has been the subject of intense activity [36], and the reactivity of probes for hypochlorous acid [44]. Earlier, a much clearer understanding of the reactions involved in the use of hydroethidine as a probe for superoxide radicals emerged after a series of careful studies [119,120], and the insightful review by Zielonka and Kalyanaraman [30] is an essential reading for users of luminescent probes for cellular oxidizing and nitrating species.

In particular, the generation of specific radicals by pulse radiolysis was used to estimate key rate constants involved in the detection of superoxide radicals by dihydroethidium (hydroethidine, HE, Figure 1, **3**), which superficially is similar to that of lucigenin in that the oxidized probe reacts with superoxide to form a detectable product. The oxidation of hydroethidine by O_2_^•−^ has a rate constant around 2 × 10^6^ M^−1^ s^−1^; the rate constant for the reaction of (HE)^•+^ with O_2_^•−^ to form the measured product, 2-hydroxyethidium, is orders of magnitude higher; so, the first reaction may be rate-limiting, depending on the concentrations of HE and (HE)^•+^—at least in systems where only O_2_^•−^ is the oxidant: obviously, other oxidants can achieve the first step, as directly observed using radiolytically produced radicals [120].

‘Amplex^®^ Red’, a dihydroxyphenoxazine (Figure 1, **4**), has been quite widely used to assay hydrogen peroxide, peroxidase-catalyzed oxidation yielding the fluorescent dye resorufin. However, other oxidants, including biological oxidants, are also reactive: stopped-flow kinetic spectrophotometry was used to demonstrate that peroxynitrite-derived oxidants, but not peroxynitrite, also oxidized the probe, and pulse radiolysis quantified the reactivity towards carbonate radicals, although at pH 10.3 (where the reactivity might be higher than at pH 7.4 because of prototropic properties that the authors demonstrated [121]). Intermediates formed during the conversion of Amplex^®^ Red into resorufin were characterized. An earlier study [10] discussed possible chain reactions.

The reactivity towards superoxide of a new mitochondria-targeted probe, the phenanthridine derivative ‘MitoNeoD’ (Figure 1, **5**), was also characterized using pulse radiolysis, with a rate constant for the reaction of the probe with O_2_^•−^ of ~1.4 × 10^7^ M^−1^ s^−1^ measured [122]. The details of this study are outside the scope of the present paper but are well worth studying as they demonstrate the depth of understanding now being applied in the development of new probes.

## 5. Some Compilations of Rate Constants for Reactions of Free Radicals Relevant to Biology

While there are very few rate constants measured for reactions of radicals with the specific chemical probes of the general type discussed here, there are useful, if dated, compilations of expertly evaluated rate constants for reactions of ^•^OH and H^•^ [123,124], HO_2_^•^/O_2_^•−^ [125], peroxyl radicals [126], inorganic radicals including CO_3_^•−^, ^•^NO_2_ and ClO_2_^•^ [103], phenoxyl radicals [127] and aliphatic carbon-centered radicals [128]. It may be possible to estimate the likely rate constants in a few instances by comparison with the simpler chemical models that have been studied, and these compilations are invaluable in considering the chemical kinetics of possible competing reactions with endogenous reactants. However, the publication dates should be noted; further, while the former database of the University of Notre Dame Radiation Chemistry Data Center (which hosted the production of these compilations) has been transferred to the website of the National Institute of Standards and Technology (see: https://kinetics.nist.gov/solution/, accessed on 23 May 2023), it too suffers from not being updated and has strict syntax. It should be noted that PubMed will often miss relevant studies of a purely chemical nature. The majority of the rate constants involving reactions of interest involving free radicals have been obtained using radiation–chemical methods, and the inclusion of the term ‘pulse radiolysis’ as a search term may help filter literature searches for relevant kinetic data in both PubMed and Chemical Abstracts (SciFinder^®^). However, in recent years several pulse radiolysis facilities in Europe have closed down, and this is a barrier to the future application of the technique in this area.

## 6. Key Points to Consider When Using Chemical Probes in Free-Radical Biology

Biochemists familiar with enzyme-catalyzed reactions will take for granted that an assay for an enzyme will involve conditions such that the catalyst is rate-limiting, rather than the concentration of the substrate(s) (or vice versa if a substrate is to be assayed using an enzyme-catalyzed assay): saturating conditions characterized by Michaelis–Menten kinetics. Yet, in attempting to assay ‘ROS’ and other biological oxidants, the necessary parameters to evaluate whether a probe is used under saturating conditions or to estimate the fraction of the oxidant that is being detected—the products of rate constants and concentrations for reactions of both probe and competing reactants—have often been ignored or are simply not available.

The applications of chemical probes vary widely, and the criteria for using them in cell-free systems may be very different from those for cellular models and especially tissues. However, it may be useful to reiterate briefly the main questions that researchers should ask *before* using a probe, based on earlier discussion [5,13,30]:

- *Where is the probe located, and what is its concentration?* Consider, if appropriate, whether there are likely to be extracellular–intracellular concentration gradients or inter-organelle or other local variations in the concentration of the probe (driven by, e.g., lipid/water partitioning, trans-membrane pH differentials or binding to macromolecules). Measure, or at least estimate, the probe concentration(s) in the region(s) of interest.
- *What are the species likely to react with the probe?* Consider *all* the putative species being generated and their reactivities towards the probe, as estimated by the product of rate constant and concentration, preferably under relevant conditions (pH; solvent polarity; ideally, also temperature, although competing reactions *may* exhibit broadly similar temperature effects).
- *What are the reaction pathways involved in the generation of the final product being measured*? Identify reaction intermediates if possible, noting that spin conservation is likely to dictate the obligate formation of intermediate free radicals from radical oxidants.
- *Is a catalyst involved in the reaction(s)?* Consider whether the concentration of the catalyst may be rate-limiting and whether the presence of the catalyst results from the treatment being assessed, e.g., the release of cytochrome *c* from mitochondria as a result of apoptosis initiated by the oxidative challenge.
- *What are possible competing reactions, firstly involving kinetic competition between the probe and cellular antioxidants for the species of interest (e.g., oxidizing radicals or molecules)?* The Law of Mass Action will dictate the extent of the competition involving reactions of the species being assessed with endogenous reactants, especially with antioxidants: thiols, ascorbate, urate, NADH, α-tocopherol, etc., and other redox-active reagents (including oxygen, which can also modulate thiol radical chemistry).
- *Do the intermediates in probe chemistry react with endogenous molecules in competition with pathways leading to the final, measured product?* Again, the Law of Mass Action is central to an analysis, and quantifying the redox properties of reactive intermediates will help suggest possible reactants. The archetypical example is the reaction with oxygen of the obligate intermediate radical obtained on oxidation of reduced fluorescein dyes—oxidation either by radical species or by intermediates formed in the catalyzed reaction with hydrogen peroxide—to *generate* superoxide radicals.
- *Are there any potential effects of the cell culture medium (if used)?* Consider comparing the results in full medium (proteins, amino acids, redox- or light-sensitive pH indicator, metals, ascorbate, etc.) with those from cells suspended/plated in as pure phosphate-buffered saline as can be obtained.
- *Could there be artefacts arising from the photochemical properties of the probes and products?* Visible light (including ambient room light or instrumental light sources) may initiate photochemical reactions; photochemically induced excited states may be much more reactive towards cellular reductants than the probes themselves. Consider whether the product build-up is sufficient to initiate inner-filter effects by absorbing a significant fraction of the incident light, e.g., in fluorescence plate readers.
- *How far is the measured product likely to diffuse from the site of interest or ‘leak’ from cells?* This will be time-dependent and, within a specific organelle, can be estimated from the likely diffusion coefficient and the Einstein–Smoluchowski equation, but trans-membrane transport should also be considered.
- *Does the probe itself affect cellular function?* Probe and/or product may bind to macromolecules or change the mitochondrial membrane potential or may initiate apoptosis and lead to potential catalysts released during the experiment (e.g., cytochrome c in apoptosis).

## 7. Conclusions

To design, synthesize and use with confidence a chemical probe for reactants of interest in free-radical biology is a daunting prospect, requiring skills spanning disciplines, and techniques and instrumentation unlikely to be found in a single laboratory. The chemical obstacles to the use of the commonest probe for ‘ROS’ have been discussed previously (and repeatedly) but were summarized here with emphasis on kinetic factors, as a pointer to some of the properties which must be considered in the rational use of probes.

Some readers may find the discussion above of the language of free-radical biology and the advice to avoid the term ‘ROS’ wherever possible to be unrealistic, pedantic and repetitive. Yet, in the view of the present author, the language is a major part of the problem. The term ‘reactive oxygen species’ may have arisen in the early days of free-radical biology, when the emphasis was on Fenton chemistry and the term simply avoided distinguishing between free hydroxyl radicals as a product of iron/hydrogen peroxide chemistry, or whether an iron-oxo species was the reactant. The index to the second (1989) edition of a standard text discussing free radicals in biology and medicine does not include ‘reactive oxygen’ or ‘ROS’ [129]. Now, though, the term and its acronym retrieved over 327,000 ‘hits’ in PubMed at the time of writing, and it would seem impossible to avoid this vague term. One is reminded of the old computer adage, ‘GIGO’: ‘garbage in, garbage out’; in the context of chemical probes for ‘ROS’, the vagueness of the input term *almost guarantees* a vague output in the use of the probes. As a group of experts recently noted [4], ‘Multiple roles of reactive oxygen species (ROS) … have led researchers unfamiliar with the complexities of ROS and their reactions to employ commercial kits and probes to measure ROS and oxidative damage inappropriately, treating ROS (a generic abbreviation) as if it were a discrete molecular entity’.

In the present article, the author has again stressed the pitfalls in the use of the commonest probe for ‘ROS’ to illustrate what can go wrong if a careful study of the literature is outweighed by the convenience of purchasing and using a commercial reagent where the manufacturer has glossed over the problems. This was followed by a brief mention of some recent studies which show that leading workers in the field *do* have a clear grasp of the problems to be addressed. Kinetic measurements in which specific chemical entities are generated and their reactions followed directly to quantify reactivity are central to the successful application of chemical probes for oxidants and radicals in biology, but the rate constants are only part of the story; the local concentrations of the probe *and possible endogenous reactants* at specific sites in the cell are equally important. It is hoped the list above of questions that users should first consider in the use of chemical probes may, at the very least, help scientists to be aware of the uncertainties which arise if these factors are ignored.

## Data Availability

No new data were created or analyzed in this study. Data sharing is not applicable to this article.
